# Pexiganan in Combination with Nisin to Control Polymicrobial Diabetic Foot Infections

**DOI:** 10.3390/antibiotics9030128

**Published:** 2020-03-20

**Authors:** Diana Gomes, Raquel Santos, Rui S. Soares, Solange Reis, Sandra Carvalho, Pedro Rego, Maria C. Peleteiro, Luís Tavares, Manuela Oliveira

**Affiliations:** 1 Centro de Investigação Interdisciplinar em Sanidade Animal (CIISA), Faculdade de Medicina Veterinária, Universidade de Lisboa, Avenida da Universidade Técnica, 1300-477 Lisboa, Portugal; dianagomes20@outlook.com (D.G.); raq.martins.santos@gmail.com (R.S.); solange.reiis25@gmail.com (S.R.); sancarva@gmail.com (S.C.); mcpelet@fmv.ulisboa.pt (M.C.P.); ltavares@fmv.ulisboa.pt (L.T.); moliveira@fmv.ulisboa.pt (M.O.); 2 Centro de Química Estrutural, Instituto Superior Técnico, Avenida Rovisco Pais, 1049-001 Lisboa, Portugal; pedrogilsenarego@hotmail.com

**Keywords:** diabetic foot ulcers, diabetic foot infections, Antimicrobial peptides, nisin, pexiganan, *Staphylococcus aureus*, *Pseudomonas aeruginosa*, biofilm, guar gum, 3D wound model

## Abstract

Diabetic foot ulcers (DFUs) are major complications of Diabetes *mellitus* being responsible for significant morbidity and mortality. DFUs frequently become chronically infected by a complex community of bacteria, including multidrug-resistant and biofilm-producing strains of *Staphylococcus aureus* and *Pseudomonas aeruginosa*. Diabetic foot infections (DFI) are often recalcitrant to conventional antibiotics and alternative treatment strategies are urgently needed. Antimicrobial Peptides (AMPs), such as pexiganan and nisin, have been increasingly investigated and reported as effective antimicrobial agents. Here, we evaluated the antibacterial potential of pexiganan and nisin used in combination (dual-AMP) to control the growth of planktonic and biofilm co-cultures of *S. aureus* and *P. aeruginosa* clinical strains, co-isolated from a DFU. A DFU collagen three-dimensional (3D) model was used to evaluate the distribution and efficacy of AMPs locally delivered into the model. The concentration of pexiganan required to inhibit and eradicate both planktonic and biofilm-based bacterial cells was substantially reduced when used in combination with nisin. Moreover, incorporation of both AMPs in a guar gum delivery system (dual-AMP biogel) did not affect the dual-AMP antimicrobial activity. Importantly, the application of the dual-AMP biogel resulted in the eradication of the *S. aureus* strain from the model. In conclusion, data suggest that the local application of the dual-AMPs biogel constitutes a potential complementary therapy for the treatment of infected DFU.

## 1. Introduction

The prevalence of Diabetes *mellitus* is increasing, affecting more than 422 million people worldwide [1]. Approximately 15% to 25% of patients with diabetes will develop diabetic foot ulcers (DFUs) in their lifetime [2] that can result in repeated hospitalizations, amputation and premature mortality. 

Close to 1 in 2 diabetes patients with a DFU are estimated to develop a diabetic foot infection (DFI) [3,4]. DFUs can become infected by a polymicrobial community of microorganisms, often producing several virulence factors, including biofilms, that are associated to wound chronicity [5]. *Staphylococcus aureus* and *Pseudomonas aeruginosa* are the predominant Gram-positive and Gram-negative pathogens detected in DFIs [6]. Both species are known for their resistance profile towards commonly used antibiotic agents [7,8,9]. The spread of multidrug-resistant bacterial strains, along with the ineffectiveness of antibiotics in eradicating biofilm-based infections, makes the development of alternative treatment strategies urgent.

Antimicrobial peptides (AMPs) have been increasingly investigated as promising therapeutic alternatives to conventional antimicrobial therapies. This family of molecules are constituted by low molecular weight proteins, discovered from both prokaryotes and eukaryotes, and are produced as part of innate immune response mechanisms against microorganisms [10]. AMPs typically exhibit broad-range antimicrobial action [11,12] and immunomodulatory properties [13,14]. In addition, these peptides also demonstrate decreased drug interaction and low toxicity [12,15].

Pexiganan is a synthetic AMP analog of magainin, that exhibits a broad-spectrum of action and acts by disrupting the bacterial cell membrane through toroidal-type pore formation [16]. Pexiganan was originally developed by Magainin Pharmaceuticals for the treatment of infected diabetic foot ulcers and was the only AMP that reached phase III clinical trials aiming at evaluating its wound healing properties towards DFI [17]. Despite the fact that pexiganan cream formulation evaluated in those trials was not able to demonstrate a clear advantage over conventional oral antibiotics [18,19] or a topical placebo control [19,20,21], it is still one of the best-studied AMPs for therapeutic purposes [16]. As pexiganan acts directly on the anionic phospholipids of the bacterial cell membrane and not on membrane receptors [22], the development of resistance is theoretically less likely to occur [23]. Given the widespread increase of microbial resistance to most other antimicrobial agents, this characteristic, in addition to its broad spectrum of antimicrobial activity, makes pexiganan an agent meriting further study in the context of DFI treatment, aiming at enhancing its antimicrobial activity. 

Nisin is an AMP naturally produced by *Lactococcus lactis* that acts predominantly against Gram-positive bacteria, exerting its antimicrobial activity by interacting with the bacterial cell wall precursor lipid II. Nisin inhibits lipid II incorporation into the peptidoglycan network and uses it as a docking molecule for subsequent pore formation [24]. Due to its broad action spectrum, heat stability, tolerance to low pH and safety profile, nisin has been widely used as a food preservative for over 60 years [25]. Notably, nisin has been shown to be effective against both planktonic cells [26,27] and against biofilms [27,28,29] of multi-drug resistant staphylococci, including strains isolated from DFI [30,31]. Thus, nisin is an AMP with the potential to complement and enhance DFI antibacterial therapies.

Several studies have demonstrated that combinations of antimicrobial molecules often reduce their individual effective concentrations and expand their action range [7]. Indeed, the efficacy of individual AMPs can be enhanced by combination with other AMPs [32,33,34] or with conventional antibiotics [32,35,36]. The combined use of pexiganan and several conventional antibiotics has been investigated as an antimicrobial enhancement strategy against microorganisms involved in several conditions such as sepsis, respiratory tract infections or skin infections [37,38], as well as DFI [39]. Nisin may also be further improved through combination with other antimicrobials or membrane-active substances [33,36,40]; however, there are no studies evaluating the combinatory effect of pexiganan and nisin or other AMPs, in the context of DFI treatment. 

Despite their potential, AMP successful delivery represents a challenge as these molecules can be degraded or inactivated before reaching their target at therapeutic concentrations [41]. Therefore, the use of an appropriate delivery system is critical [41,42]. Natural polysaccharides have been considered promising drug delivery systems, mainly because of their non-toxicity, biodegradability, biocompatibility, abundant availability in nature and economical cost [43]. Guar gum, a natural polysaccharide consisting of galactomannan [42], has been considered a safe and versatile system for delivery of bioactive agents, including antibiotics [42] and AMPs [30,31].

The use of bioengineered platforms or model systems that mimic the infected wound state have been essential to evaluate and develop effective therapeutic approaches [44]. These models aim to provide an imitation of the chronic wound infection, including key microenvironment components such as structure, dimensionality and architecture. Given that the wound bed extracellular matrix plays an important role in wound healing and infection outcome [45], several infection models incorporate these matrix elements, focusing largely on collagen as the main component [46,47]. Collagen wound models have been employed as a substrate for *S. aureus* and *P. aeruginosa* biofilm attachment and to evaluate their susceptibility to conventional antibiotics [46]. With appropriate adaptations, these types of models may constitute a valuable tool to assess bacterial susceptibility to AMPs and to evaluate delivery systems, such as the guar gum biogel.

Here, we assessed the efficacy of a nisin and pexiganan dual-AMP biogel to control the growth of *S. aureus* and *P. aeruginosa* DFI clinical strains and established that this dual-AMP biogel exhibits increased antimicrobial activity in comparison with a pexiganan biogel and is able to eradicate *S. aureus* in a DFI collagen three-dimensional (3D) model.

## 2. Results

### 2.1. Planktonic Bacteria Susceptibility to Pexiganan And Pexiganan-Nisin Biogel

The minimum inhibitory concentration (MIC) and minimum bactericidal concentration (MBC) for pexiganan were determined regarding *S. aureus* and *P. aeruginosa* mono and dual species planktonic cultures, and compared to those from pexiganan combined with nisin (dual-AMP), either in water solution or incorporated in a guar-gum biogel (dual-AMP biogel).

**Figure 1:**
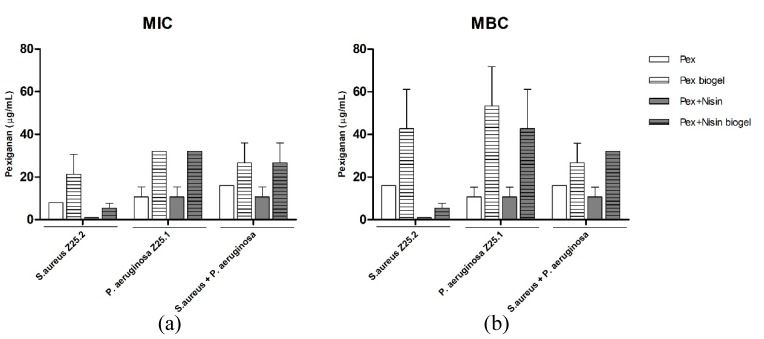
Minimum inhibitory concentrations (MIC) (**a**) and minimum bactericidal concentrations (MBC) (**b**) of pexiganan solution, pexiganan incorporated within guar-gum biogel, pexiganan and nisin dual antimicrobial peptide (AMP) solution and pexiganan and nisin dual-AMP biogel against *S. aureus* Z25.2, *P. aeruginosa* Z25.1 and dual-species planktonic cultures of these strains. Bars represent means ± standard deviation.

Pexiganan’s MICs and MBCs against *S. aureus* and *P. aeruginosa* DFI strains in dual-species suspensions was found to be up to two-fold higher than those for single species suspensions. Thus, both *S. aureus* and *P. aeruginosa* single- and dual-species planktonic cultures exhibited comparable susceptibilities to pexiganan. When pexiganan was used incorporated within a guar-gum biogel, pexiganan kept its antimicrobial activity. Pexiganan biogel MIC and MBC values against both single- and dual-species cultures were only two- to four-fold higher than the values exhibited by pexiganan diluted in water (Figure 1).

When pexiganan was used in combination with nisin, we observed an increased antimicrobial activity of this dual-AMP suspension against *S. aureus* planktonic cultures, when compared to pexiganan used alone. This effect was evidenced by a more than eight-fold reduction of MIC and MBC values. However, regarding *P. aeruginosa* and *P. aeruginosa* plus *S. aureus* dual-species planktonic cultures, the use of the dual-AMP had no impact when compared to pexiganan used alone (Figure 1).

Regarding the dual-AMP biogel formulation, the antimicrobial activity enhancement due to the presence of nisin against *S. aureus* strains was maintained. Nisin contributed to reduce pexiganan’s MIC and MBC values against *S. aureus* up to eight-fold. Regarding *P. aeruginosa* and dual-species suspensions, the pexiganan’s MIC and MBC values when used in a dual-AMP biogel were similar to those exhibited by pexiganan alone, showing that the addition of nisin to a pexiganan biogel has no relevant effect in the susceptibility of *P. aeruginosa* and dual-species planktonic cultures.

### 2.2. Biofilm Bacterial Susceptibility to Pexiganan and Pexiganan-Nisin Biogel

To investigate the anti-biofilm activity of pexiganan and of both pexiganan and nisin in solution or incorporated in a guar-gum biogel, the minimum biofilm inhibitory concentration (MBIC) and minimum biofilm eradication concentration (MBEC) of these formulations were determined.

**Figure 2:**
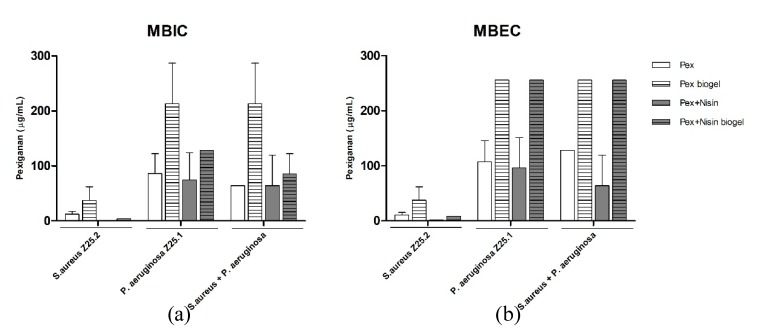
Minimum biofilm inhibitory concentrations (MBIC) (**a**) and minimum biofilm eradication concentrations (MBEC) (**b**) of pexiganan solution, pexiganan incorporated within guar-gum biogel, pexiganan and nisin dual-AMP solution and pexiganan and nisin dual-AMP biogel against *S. aureus* Z25.2, *P. aeruginosa* Z25.1 and dual-species biofilm cultures. Bars represent means ± standard deviation.

Pexiganan exhibited lower MBIC and MBEC values against biofilms formed by *S. aureus* when compared to biofilms formed by *P. aeruginosa* or to dual-species biofilms. Thus, a high inhibitory activity of pexiganan towards *S. aureus* biofilms was observed when compared to its activity against *P. aeruginosa* or dual-species biofilms. Moreover, the incorporation of pexiganan within a guar-gum biogel conserved the higher inhibitory activity of this AMP towards *S. aureus* biofilms, as compared to *P. aeruginosa* or dual-species biofilms (Figure 2).

When pexiganan was combined with nisin, we similarly observed lower MBIC and MBEC values against biofilms formed by *S. aureus*, as compared to biofilms formed by *P. aeruginosa* or dual-species biofilms. This reveals that the combination of pexiganan with nisin also exhibited an increased antimicrobial activity against *S. aureus* biofilms as compared to *P. aeruginosa* or dual-species biofilms.

When the dual-AMP was delivered in a biogel, it maintained the lower MBIC and MBEC values towards *S. aureus* as compared to biofilms formed by *P. aeruginosa* or dual-species biofilms. Moreover regarding *P. aeruginosa* and dual-species biofilms, the MBIC values of the dual-AMP biogel decreased when compared to pexiganan biogel. However, MBEC values were similar for both the dual-AMP biogel and pexiganan biogel. Thus, the addition of nisin to a pexiganan biogel increased the biofilm inhibitory activity of the biogel regarding both *S. aureus and P. aeruginosa* single-species and dual-species biofilms but regarding eradication activity, addition of nisin only increased the biogel activity against *S. aureus* biofilms.

### 2.3. Antimicrobial Biogel Difusion in a DFU 3D Model 

To assess the antimicrobial and bacterial distribution ability across a DFU 3D model, the diffusion of pexiganan and nisin when incorporated in the guar gum biogel was evaluated. 

When nisin-biogel was administered to the model in a single application followed by a 24 h incubation, the AMP diffused through the area 1 and area 2 of the model but it was not detected in the area 3, as inferred by the formation of inhibition halos in cultures exposed to samples taken from each area of the model (Table 1). 

Similarly, pexiganan-biogel diffused through the areas 1 and 2 of the model. However, no antibacterial activity was detected in the area 3, when pexiganan was administered in a single application followed by a 24 h incubation (Table 1).

### 2.4. Bacterial Difusion in a DFI 3D Model

For bacterial distribution through the DFI 3D model, a DFU was also assessed by inoculating the model with *S. aureus* or *P. aeruginosa* single and dual cultures, followed by incubation and bacterial quantification in the different areas of the model.

*S. aureus* and *P. aeruginosa* single-species cultures were able to diffuse across all areas of the collagen 3D model. When *S. aureus* and *P. aeruginosa* were inoculated as a dual-species inoculum, both species were detected in the three areas of the collagen model in similar bacterial concentrations (Table 2). 

**Figure 3:**
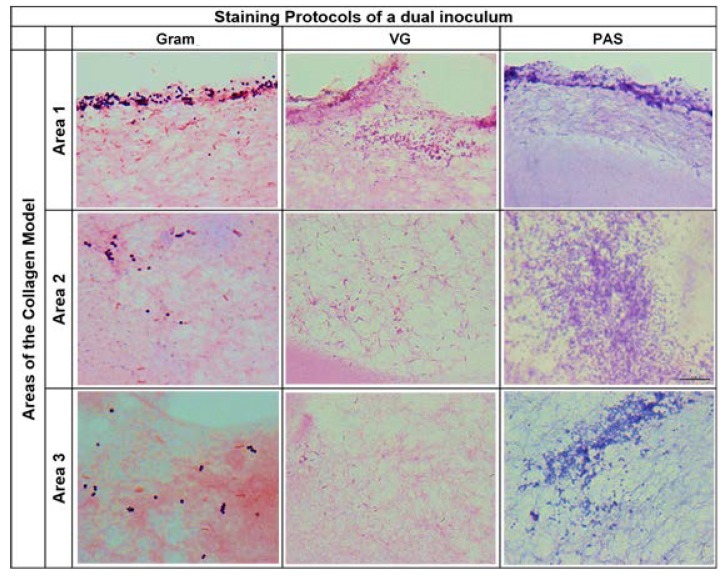
Evaluation of *S. aureus* Z 25.2 and *P. aeruginosa* Z 25.1 presence in the 3D model after inoculation with dual inoculums. Gram-staining revealed Gram-positive bacteria stained in purple and Gram-negative bacteria stained in pink, corresponding respectively to *S. aureus* Z 25.2 and *P. aeruginosa* Z 25.1. Van Gieson (VG) staining revealed the collagen matrix in light pink and bacteria stained in a darker pink. Periodic Acid-Schiff (PAS) staining revealed the collagen matrix in purple and the polysaccharide matrix stained in a darker purple. Area 1 corresponds to the inner circular area with 1.5 cm diameter; Area 2 corresponds to the circular area between 1.5 cm and 2 cm diameter; Area 3 corresponds to the circular area with a diameter of >2 cm. All images were obtained from 10 µm sections of the same paraffin blocks (Original, 1000x).

These results were confirmed by microscopic analysis using the Van Gieson (VG) and Gram-staining protocols, allowing for observation of the presence of *S. aureus* and *P. aeruginosa* in the three areas of the collagen DFI 3D model, both when bacterial strains were inoculated as single-species or as dual-species inoculum. Moreover, the presence of biofilm extracellular matrix was also confirmed using the Periodic Acid-Schiff (PAS) staining protocol (Figure 3).

### 2.5. Pexiganan and Pexiganan-nisin Dual-AMP Biogel Inhibitory Activity in a DFI 3D Model

The DFI 3D model was used to test the inhibitory activity of the pexiganan-biogel and the dual-AMP biogel against *S. aureus* and *P. aeruginosa* inoculated in the model. 

The application of the pexiganan-biogel in the DFI 3D model demonstrated an increased antimicrobial activity against *S. aureus* as compared to *P. aeruginosa*. Pexiganan-biogel was able to decrease *S. aureus* bacterial concentration in all areas of the model, causing a ten- to twenty-fold decrease in bacterial concentration (Table 3). Regarding *P. aeruginosa*, the pexiganan-biogel caused a decrease in the bacterial concentration from area 1 to area 2 of the inoculated model, that was not maintained in area 3. Nevertheless, pexiganan was able to decrease *P. aeruginosa* concentration in all areas of the model. 

When nisin was combined with pexiganan in a dual-AMP biogel, strong antibacterial activity against *S. aureus* was observed, resulting in the eradication of the *S. aureus* isolate in all areas of the collagen DFI 3D model. Regarding *P. aeruginosa*, the dual-AMPs exhibited limited inhibitory activity against this isolate. The *P. aeruginosa* was detected across all areas of the model and no significant reduction in bacterial concentrations were detected.

## 3. Discussion

Currently, the management of DFIs includes debridement and antibiotherapy [5,6]. However, the emergence of antibiotic-resistant strains and their propensity to form recalcitrant biofilms, often render this approach inefficient [5], highlighting the urgency in the development of new treatment strategies to effectively eradicate these infections [48].

Here, we set out to examine for the first time the ability of pexiganan combined with nisin incorporated in a guar gum biogel, to control *S. aureus* and *P. aeruginosa* clinical strains co-isolated from the same DFU, aiming to evaluate the potential of this dual-AMP biogel to treat polymicrobial DFIs. Following MIC, MBC, MBIC and MBEC determinations of the dual-AMP biogel, an increased antibacterial activity was observed against both planktonic and biofilm cultures of *S. aureus* and against dual cultures of *S. aureus* and *P. aeruginosa*. Moreover, this dual-AMP biogel was able to eradicate *S. aureus* biofilms from the collagen matrix of a DFI 3D model. These results reveal that supplementation of pexiganan with nisin at MIC levels can effectively control the growth of *S. aureus* and *P. aeruginosa* DFI clinical strains. In contrast, pexiganan used independently was unable to eradicate *S. aureus* and exhibited a lower antimicrobial activity against *P. aeruginosa* biofilms when compared to the dual-AMP biogel. 

It has been reported that pexiganan exhibits a broad in vitro antimicrobial activity spectrum against most of the common pathogens isolated from infected diabetic foot ulcers [49]. Moreover, to date, pexiganan is the only AMP reaching a phase III clinical trial aiming at the treatment of DFIs [20]. However, the topical formulation evaluated in those studies was not able to demonstrate superior antimicrobial activity when compared to conventional oral antibiotics or a topical placebo [20], suggesting that pexiganan used alone might not be sufficient to eradicate DFI biofilms in a clinical context. Regarding pexiganan antimicrobial activity against planktonic cultures, our results showed that, when used alone, it is similarly effective against *S. aureus* and *P. aeruginosa*, in agreement with previous studies [49,50]. As the difference between MICs and MBCs has been established as an index of the bactericidal activity of an antibiotic [51], the similar MICs and MBCs of pexiganan against *S. aureus* and *P. aeruginosa* DFI strains that were observed in this study are consistent with the bactericidal mechanism of action of pexiganan [7]. 

It has been demonstrated that the combination of AMPs can enhance the antibacterial properties of each compound as compared to its separate use [32,33]. AMPs can act in synergy with conventional antibiotics, particularly when they exhibit different mechanisms of action [52]. Recent studies have demonstrated that AMP can enhance the activity of antibiotics, antifungals and other antimicrobials when used in combination [7]. Here, we demonstrated that the combination with nisin allowed to reduce the concentration of pexiganan required to inhibit and eradicate the DFI isolates, either in their planktonic or biofilm states. This effect was more noticeable on *S. aureus* monocultures than on *P. aeruginosa* ones, which is probably related with nisin’s mode of action. Upon binding to lipid II, nisin inhibits cell wall biosynthesis and promotes the formation of pores in bacterial membranes, leading to cytoplasmic constituents’ efflux and cell death [24]. Considering that lipid II is mainly located at the inner membrane, the outer membrane of Gram-negative bacteria may prevent nisin from reaching lipid II molecules, rendering Gram-positive bacteria more susceptible to nisin than Gram-negative ones [53]. Moreover, the ability of nisin to form stable pores on prokaryotic cell membranes has also been suggested for biofilm-based bacteria [27,30], possibly explaining its potent antimicrobial activity not only against *S. aureus* planktonic cultures but also against biofilms. 

The ability of nisin to complement pexiganan’s anti-biofilm activity favors their combined use in the treatment of recalcitrant DFIs. AMPs such as nisin and pexiganan, known to disrupt the bacterial membrane, might be good adjuvants for antibiotics that target bacterial intracellular pathways. Therefore, the use of this novel dual-AMP biogel may potentially benefit future novel multifactorial approaches towards DFI treatment.

Given that bacterial infections in a DFU are frequently growing in the form of a biofilm [54], conventional antibiotics would have increased MIC and the administration of standard therapeutic concentrations may not be relevant in these infections, contributing to the high rate of nonhealing ulcers in DFI [55,56]. Indeed, it has been estimated that biofilm-based bacteria can tolerate antimicrobial agents at concentrations 10 to 1000 times higher than their genetically equivalent planktonic forms [57]. In these cases, local application of antimicrobial may deliver high concentrations, allowing a close interaction between the antimicrobial and the biofilm and avoiding the problems related with systemic use of antimicrobials [58]. Few studies have investigated the effect of locally applied antimicrobials in DFI and ultimately, its effects in wound closure [59]. Some studies have shown promising results such as using pexiganan cream [39], gentamicin incorporated into a collagen implant [60], tobramycin-impregnated calcium sulfate pellets [61] or in nanofibers and nanomembranes [62]; however, there is no support for some of these therapies by the regulatory agencies because they did not met the principal purpose of complete closure of the wounds [48].

Our data shows that when a guar gum biogel was used as a delivery system for the pexiganan and nisin dual-AMP, an increased antibacterial activity against both planktonic and biofilm cultures of *S. aureus* and against dual cultures of *S. aureus* and *P. aeruginosa* was observed. Results also confirmed the guar gum biogel potential as a delivery system for these AMPs. Indeed, we have previously shown that the natural polysaccharide guar gum displayed very good efficacy as a delivery system for nisin, as its antimicrobial activity toward *S. aureus* DFU strains was kept when incorporated in a guar gum biogel [30].

In order to further study the applicability of this dual-AMP biogel towards DFI treatment, we developed a DFI collagen 3D model, aiming at better mimic the in vivo conditions of a DFU. The collagen wound model allowed us to confirm the distribution of DFI *S. aureus* and *P. aeruginosa* clinical strains along the ulcer model as well as the diffusion of pexiganan and nisin through a physiologically relevant substrate and to validate the effects of the dual-AMP biogel on the development of bacterial biofilms. Our data shows that the two AMPs present in the dual-AMPs biogel were able to effectively diffuse and maintain their antimicrobial activity against biofilm-embedded bacteria in the collagen DFI 3D model. The application of the supplemented guar gum gel in the 3D model, with 8 hour intervals during a 24 hour period, aimed to mimic the ulcer treatment protocols applied at the clinical settings, where the dressings are changed frequently, depending on the DFU severity [63]. Despite the model limitations, as it is a closed system and misses other factors that may occur in a DFI, such as the presence of immune cells, the presence of exudate or wound drainage, it is probably closer to the in vivo situation than other models based on poly(methyl-methacrylate) (PMMA) disks or cellulose and agar plate zone inhibition assays. Also, the biofilm growth in a 3D collagen matrix naturally mimics the limited oxygen availability in a DFU. The use of a nutrient-limited medium, like simulated wound fluid (SWF), may also contribute to a more robust biofilm formation than the medium used in conventional MBEC assays. Thus, these types of models might be useful to further study possible enhancement or complementation properties of this AMP-biogel when used in combination with conventional antibiotics or other experimental antibacterial molecules.

Given that pexiganan in combination with nisin in a dual-AMP biogel formulation have shown a strong inhibitory and eradication effect against DFI *S*. *aureus* biofilms, we propose that this antimicrobial combination could complement DFI antibiotherapy, possibly enhancing conventional antibiotics activity and potentially contribute to reduce the burden of antibiotic-resistant infections. Therapeutic protocols that include a topical application of AMPs may represent a promising approach to complement the treatment of chronically infected DFUs, potentially contributing to the reduction of antibiotic administration, selection pressure on DFI pathogens and dissemination of resistance strains. 

## 4. Materials and Methods 

### 4.1. Bacterial Isolates

In this study, we used the biofilm-producing DFI strains *Staphylococcus aureus* Z25.2 and *Pseudomonas aeruginosa* Z25.1, co-isolated from a diabetic foot ulcer. The strains were selected from a bacterial collection obtained from patients with Diabetes *mellitus* and infected foot ulcers, as described in a previous study by Mendes et al. [6] and characterized by Mottola et al. [64]. All isolates were stored at –80 °C in buffered peptone water supplemented with 20% (*v/v*) of glycerol. When needed, cells were streaked and grown on Mueller Hinton Cation-Adjusted (MH-CA) agar medium (Becton, Dickinson and Company, USA) at 37 °C for 24 h. Bacterial suspensions were prepared at 10^8^ cfu/mL directly from plate cultures using a 0.5 McFarland standard (BioMérieux) in sterile normal saline (Scharlau) and diluted in fresh MH-CA broth to obtain 10^7^ cfu/mL suspensions for minimum inhibitory concentration (MIC) and minimum bactericidal concentration (MBC) assays, and of 10^6^ cfu/mL for minimum biofilm inhibitory concentration (MBIC) and minimum biofilm eradication concentration (MBEC) assays. Dual-microbial suspensions containing equal concentrations of each pathogen were also prepared.

### 4.2. Antimicrobial Peptides (AMPs)

A stock solution of nisin was prepared at 1000 µg/mL by dissolving nisin (1000 UI/mg, 2.5% purity) (Sigma-Aldrich, USA) in 0.02 M HCl (Merck, Germany). A pexiganan stock solution was prepared at 2048 µg/mL by dissolving pexiganan (>95% purity, Innovagen, Sweden) in deionized sterile water. Both nisin and pexiganan stock solutions were filtered using a 0.22 µm filter (Millipore Corporation, Billerica, MA, US) and stored at 4 °C. Working solutions of pexiganan were diluted in water or incorporated within the biogel at concentrations ranging from 1 to 256 µg/mL and incorporated in the collagen model at 256 µg/mL. The dual-AMPs solutions were prepared by supplementing pexiganan solutions with nisin at MIC values, as determined in a previous study by Santos et al. [30]. Briefly, nisin was added at 12.5 µg/ mL for the dual-AMP suspension in water and at 22.5 µg/mL for the dual-AMP incorporated within the biogel. Working solutions of nisin were diluted in water at a concentration of 12.5 µg/mL, incorporated within the biogel at 22.5 µg/mL and used in the collagen model at 125 µg/mL.

### 4.3. Guar Gum Biogel

The guar gum biogel was prepared as previously described by Santos et al. [30]. Briefly, a guar gum gel of 1.5% (w/v) was prepared by dissolving 0.75 g of guar gum (Sigma-Aldrich, USA) in 50 mL of sterile distilled water and heat sterilized by autoclave. AMPs dilutions were incorporated within the guar gum gel in a proportion of 1:1.

### 4.4. Planktonic Cultures Susceptibility to Single and Dual-AMPs Suspensions

The effect of AMP on planktonic cultures was evaluated by the determination of the minimum inhibitory concentration (MIC) and the minimum bactericidal concentration (MBC).

Serial dilutions of pexiganan or pexiganan and nisin dual-AMP suspensions, either in solution or incorporated within the biogel, were distributed in flat bottom 96-well polystyrene microtiter plates (Nunc; Thermo Fisher Scientific). The wells were then inoculated with 150 μL of *S. aureus* or *P. aeruginosa* bacterial suspensions at a concentration of 10^7^ cfu/mL, prepared as described in Section 4.1. The microplates were statically incubated at 37 °C for 24 h. MIC values were established as the lowest concentration of AMP that visually inhibited the bacterial growth. MBC were determined by inoculating 3 μL from each well with no visible bacterial growth in brain heart infusion (BHI) agar plates. The plates were then incubated at 37 °C for 24 h and the MBC values were established as the lowest concentrations at which no bacterial colonies were observed. 

### 4.5. Biofilm Susceptibility to Single- and Dual-AMP Suspensions

The effect of AMP on biofilm cultures was evaluated by the determination of the minimum biofilm inhibitory concentration (MBIC) and the minimum biofilm eradication concentration (MBEC).

Biofilms were developed on hydroxyapatite-coated pegs on the lid of a 96-well microplate (MBEC Biofilm Inoculator; Innovotech, Canada). The minimum biofilm inhibitory concentration (MBIC) and minimum biofilm eradication concentration (MBEC) assays were adapted from the protocol previously described by Santos et al. [30]. Briefly, bacterial suspensions were diluted in fresh tryptic soy broth (TSB) (VWR Chemicals) with 0.25% (w/v) glucose (Merck) medium to a concentration of ~10^6^ cfu/mL. 200 μL of the bacterial suspensions were transferred to 96-well flat-bottomed polystyrene microtiter plates, covered with 96-peg polystyrene lids (Nunc-TSP; Thermo Fisher Scientific) and statically incubated for 24 h at 37 °C, to allow biofilm formation on the pegs. Peg lids were then rinsed and placed on new microplates containing 200 µl of fresh TSB + 0.25% glucose medium and the set of AMP concentrations, prepared as described in Section 4.2. Microplates were incubated for 24 h at 37 °C without shaking. After incubation, peg lids were removed, and the MBIC value was determined as the lowest AMP concentration that visually inhibited the microbial growth.

For the determination of MBEC values, peg lids were rinsed three times in sterile normal saline, placed in new microplates containing 200 µl of fresh TSB + 0.25% (w/v) glucose medium and incubated in an ultrasound bath (Grant MXB14), at 50 Hz for 15 min in order to disperse the biofilm-based bacteria from the peg surface. Afterwards, peg lids were discarded, and microplates were covered with normal lids and incubated for 24 h at 37 °C. MBEC was determined through direct observation of experimental wells and defined as the lowest AMP concentration that visually eliminates the microbial growth. 

### 4.6. Collagen DFI 3D Model

A closed-system collagen 3D model was built to compare the dissemination of the AMPs, bacteria and assess the susceptibility of *S. aureus* and *P. aeruginosa* to AMP in a setting aiming at mimicking a soft-tissue collagen matrix. 

The collagen DFI 3D model setting was based on previous studies by Werthén et al. [46] and Price et al. [47], with modifications. Briefly, the system was composed of 6-well plates (Corning; Falcon, USA), to which transwell inserts (3.0 µm pore size, HD PET Membrane Permeable Supports; Corning, Falcon; USA) were placed and used as a mold for polymerization of a collagen solution containing 25% (*v/v*) of Collagen I High Concentration from rat tail (8.24 mg/mL, Corning, US), 50% (*v/v*) of cold Simulated Wound Fluid (SWF) and 25% of NaOH 0.1M (Merck, Germany) with pH adjusted to 7.5. The SWF was composed of 50% fetal bovine serum (Biowest; France) and 50% of peptone water (Biokar Diagnostics; France). For each insert, 8 mL of collagen solution was used. Afterwards, a sterilized peg-lid, specifically designed for this assay, was used as a mold to create a void on the center of each collagen insert, aiming at mimicking a DFI ulcer. The peg-lid was placed on the plate and the setting was incubated at 37 °C for 90 minutes in a humid chamber for collagen polymerization. The diameter of the voids created by the peg-lid was 12 mm, that is comparable to the size of a grade 1B ulcer [65].

### 4.7. Assessment of AMP Difusion in the DFI 3D Model

**Figure 4:**
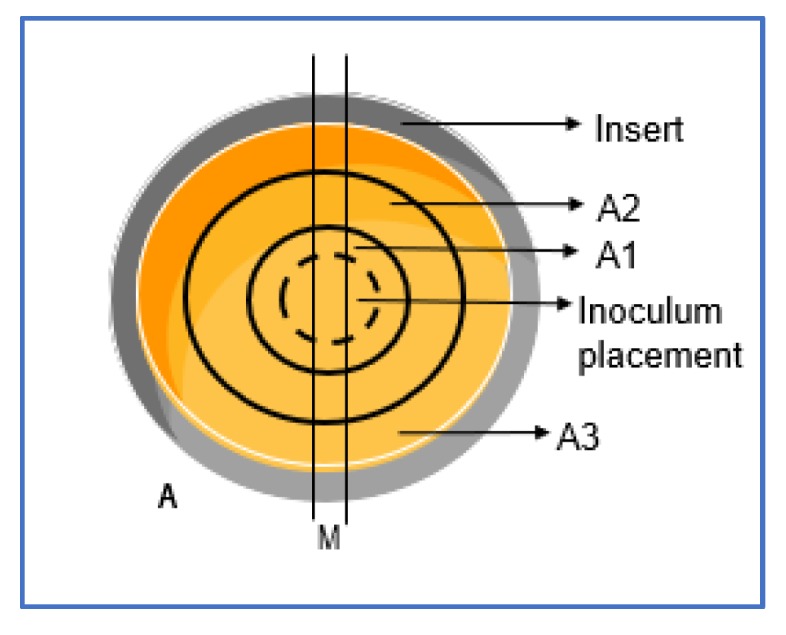
Scheme of the sectioning procedure of the collagen DFI 3D model for AMP and bacterial diffusion assessment. (**A**) Top view of the collagen 3D model in the insert, with the black lines representing the sectioned areas. (A1) Area 1, obtained with a glass tube with a diameter of 1.5 cm. (A2) Area 2, obtained with a glass tube with a diameter of 2 cm. (A3) Area 3, with diameter of >2cm. (M) Section used for microscopic analysis.

Before starting the assay, it was necessary to confirm AMPs ability to diffuse in the DFI model. Following polymerization of the collagen 3D model, 5 mL of SWF, prepared as described in Section 4.6., were transferred to each well. Afterwards, 2 mL of each AMP solution were added to the void and the model was kept at 37 °C for 24 hours. The quantification of each antimicrobial solution was performed in three distinct areas in the model (Figure 4), sectioning the collagen using sterile glass tubes with different diameters. The total collagen volume from each area was collected into 15 mL tubes and digested using 1 mL of collagenase solution (500 µg/mL in PBS; Merck, Germany), followed by incubation at 37 °C for 90 minutes, with homogenization by vortex every 30 minutes [47]. Collagen suspensions were centrifuged at 4000x g for 10 minutes at 4 °C to obtain the supernatant [46]. Afterwards, 20 µl of the supernatant correspondent to each area were placed in trypticase soy agar (TSA) plates containing a 1 × 10^7^ cfu/mL bacterial lawn of *S. aureus* Z 25.2 and/or *P. aeruginosa* Z 25.1 (according to the performed assays). Finally, the plates were incubated at 37 °C for 24 h, after which the presence of inhibition halos were assessed, and the respective diameter was measured and compared with those promoted by fixed AMP concentrations. The experiments were performed in duplicate.

### 4.8. Assessment of Bacterial Difusion in the DFI 3D Model

Following polymerization of the collagen 3D model, 5 mL of SWF were added to each well together with 500 µL of bacterial suspensions at 1 × 10^6^ cfu/mL prepared in SWF. The 6-well plates were then incubated at 37 °C for 24 h. Afterwards, the collagen model was sectioned (Figure 4) and the total collagen volume from each area was collected into 15 mL tubes, digested, and a pellet was obtained by centrifugation at 4000x g for 10 minutes at 4 °C. The pellet was resuspended in 1 mL 0.9% NaCl and was 10-fold serial diluted. 100 µl of each bacterial dilution were inoculated in tryptone soy agar plates, followed by incubation at 37 °C for 24 h, with posterior colony counts. Experiments were performed for the bacterial strains *S. aureus* Z 25.2 and *P. aeruginosa* Z 25.1 and for their dual bacterial suspension, in duplicate, according to the performed assays.

**Figure 5:**
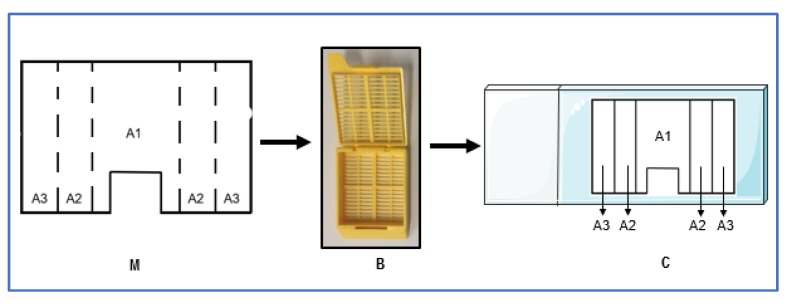
Scheme of the (M) section area used for the bacteria diffusion analysis through microscopy. (A1) Area 1, corresponding to the inner circle with 1.5 cm diameter; (A2) Area 2, corresponding to the disc between 1.5 cm and 2 cm diameter; (A3) Area 3, corresponding to the disc with a diameter of >2 cm. (B) Cassette used to process the sectioned area. (C) Placement of the sectioned area in the slide.

Bacterial diffusion and the presence of biofilm extracellular matrix in the collagen 3D model was also evaluated by microscopic analysis. The model was inoculated with the *S. aureus* Z 25.2 and/or *P. aeruginosa* Z 25.1 and incubated as described above. The insert with the collagen model was placed in a sterile flask containing 4% buffered aqueous solution of formaldeyde (VWR Chemicals; Belgium). Then, a cross-section of the model was performed and placed in a cassette (Cassettes Microstar III, VWR; France) for routine processing as a regular tissue. After processing, 10 µm sections were performed from the paraffin block containing the collagen model. The sections were placed into adhesive slides (Thermo Scientific, Sweden) (Figure 5) and stained using Van Gieson (VG), Periodic Acid-Schiff (PAS) and Gram. The slides were then visualized in an Olympus BX51 microscope, at 1000x magnification. The images were obtained with an Olympus DP21 camera. 

### 4.9. Assessment of the Inhibitory Activity of Antimicrobial Biogel in the DFI 3D Model

After the polymerization of the collagen model, 5 mL of SWF were added to each well and 500 µL of *S. aureus* Z 25.2, *P. aeruginosa* Z 25.1 and a dual bacterial suspension with 1 × 10^6^ cfu/mL of each strain prepared in SWF were inoculated in the model. Then, the plate was incubated at 37 °C for 24 h in a humid chamber to allow bacteria diffusion. After incubation, 2 mL of each antimicrobial solution were added to the insert, following incubation at 37 °C during 8 h in a humid chamber. Then, 2 mL of an antimicrobial solution were added to the insert, after removing 1 mL of the inoculated SWF present in the well, that was used for bacterial quantification and incubated at 37 °C for 8 h. This process was repeated one more time, in order to achieve a total incubation period of 24 h. After incubation, bacterial quantification was performed, in duplicate, as previously described. According with the performed assays, the antimicrobial biogel was supplemented with nisin, pexiganan or with both AMPs.

## 5. Conclusions

The antimicrobial peptide nisin, used in combination with pexiganan and delivered through a guar gum biogel, can reduce the concentration of pexiganan required to inhibit and eradicate established biofilms formed by DFI isolates, particularly of staphylococcal origin. Our results further support the use of guar gum biogel as a delivery system for antimicrobial compounds and suggest that pexiganan, in combination with nisin in a dual-AMP biogel, may constitute a potential topical antimicrobial to complement conventional antibiotic therapy applied for the treatment of diabetic foot infections. 

## Figures and Tables

**Table 1:** Quantification of Nisin and Pexiganan diffusion through the collagen diabetic foot infections (DFU) three-dimensional (3D) model.

AMP Biogel	AMP Quantification	Area 1 ^3^	Area 2 ^3^	Area 3 ^3^
**Nisin**	Ø halos ^1^	2.8 ± 0.35	2.0 ± 0	0
	(AMP) ^2^	1.85	1.10	0
**Pexiganan**	Ø halos ^1^	4.0 ± 0	3.5 ± 0.71	0
	(AMP) ^2^	200.09	184.0	0

^1^ Inhibition zone diameter halos (diameter in mm with standard deviation); ^2^ (AMP): AMP concentration in µg/mL; ^3^ Areas of the model: Area 1 has a 1.5 cm diameter; Area 2 has a 2 cm diameter; Area 3 has a diameter of >2 cm.

**Table 2:** Quantification of bacterial diffusion through the collagen DFI 3D model 24 h after single or dual species inoculums.

Inoculum Type	Bacterial Species	Area 1 ^3^	Area 2 ^3^	Area 3 ^3^
Single inoculum ^1^	*S. aureus* Z 25.2	2.3 × 10^7^	3.1 × 10^7^	3.1 × 10^7^
	*P. aeruginosa* Z 25.1	2.0 × 10^8^	1.0 × 10^10^	5.7 × 10^9^
Dual inoculum ^2^	*S. aureus* Z 25.2	5.5 × 10^7^	5.0 × 10^7^	2.8 × 10^9^
	*P. aeruginosa* Z 25.1	3.5 × 10^7^	5.5 × 10^7^	2.0 × 10^9^

^1^ Inoculation of 1.0 × 10^6^ of either *S. aureus* Z 25.2 or *P. aeruginosa* Z 25.1 in the collagen model; ^2^ Inoculation of 1.0 × 10^6^ of both *S. aureus* Z 25.2 and *P. aeruginosa* Z 25.1 in the collagen model. ^3^ Areas of the model: Area 1 has a 1.5 cm diameter; Area 2 has a 2 cm diameter; Area 3 has a diameter of >2 cm. Data are mean results of bacterial concentrations in cfu/mL.

**Table 3:** Quantification of AMP inhibitory activity in the DFI 3D model.

AMP Biogel	Bacterial Strains	Before AMP addition	Area 1 ^3^	Area 2 ^3^	Area 3 ^3^
**Pexiganan** ^1^	*S. aureus* Z 25.2	2.2 × 10^8^	7.5 × 10^6^	1.3 × 10^7^	1.5 × 10^6^
	*P. aeruginosa* Z 25.1	5.0 × 10^8^	1.7 × 10^8^	9.0 × 10^7^	3.3 × 10^8^
**Dual-AMP** ^2^	*S.aureus* Z 25.2	5.2 × 10^7^	0	0	0
	*P. aeruginosa* Z 25.1	3.0 × 10^8^	3.6 × 10^7^	1.1 × 10^8^	1.1 × 10^8^

^1^ Pexiganan added to the collagen model at 256 µg/mL; ^2^ Pexiganan and nisin added to the collagen model at 256 µg/mL and 125 µg/mL, respectively. ^3^ Areas of the model: Area 1 has a 1.5 cm diameter; Area 2 has a 2 cm diameter; Area 3 has a diameter of >2 cm. Data are mean results of bacterial concentrations in cfu/mL.
